# 2-Methyl-3-(2-methyl­phen­yl)-4-oxo-3,4-dihydro­quinazolin-8-yl thio­phene-2-carboxyl­ate

**DOI:** 10.1107/S1600536812006459

**Published:** 2012-02-17

**Authors:** Adel S. El-Azab, Alaa A.-M. Abdel-Aziz, Seik Weng Ng, Edward R. T. Tiekink

**Affiliations:** a Department of Pharmaceutical Chemistry, College of Pharmacy, King Saud University, Riyadh 11451, Saudi Arabia; b Department of Organic Chemistry, Faculty of Pharmacy, Al-Azhar University, Cairo 11884, Egypt; c Department of Medicinal Chemistry, Faculty of Pharmacy, University of Mansoura, Mansoura 35516, Egypt; d Department of Chemistry, University of Malaya, 50603 Kuala Lumpur, Malaysia; e Chemistry Department, Faculty of Science, King Abdulaziz University, PO Box 80203 Jeddah, Saudi Arabia

## Abstract

In the title compound, C_21_H_16_N_2_O_3_S, the central quinazolin-4-one ring is planar (r.m.s. deviation = 0.037 Å). The *N*-bound benzene and thio­phenyl rings are almost perpendicular to the central plane [dihedral angles = 82.22 (5) and 77.05 (13)°, respectively]. Mol­ecules are connected into a three-dimensional array by C—H⋯O inter­actions involving both carbonyl O atoms. The thio­phene ring is disordered over two positions, which are approximately parallel and oppositely orientated. The major component refined to a site-occupancy factor of 0.6555 (17).

## Related literature

For the pharmacological activity of substituted quinazolin-4(3*H*)-ones, see: El-Azab & El-Tahir (2012 ▶); El-Azab *et al.* (2010 ▶, 2011 ▶); Al-Omary *et al.* (2010 ▶); Al-Obaid *et al.* (2009 ▶); Aziza *et al.* (1996 ▶). For the synthesis and evaluation of the anti-convulsant activity of the title compound, see: El-Azab *et al.* (2010 ▶).
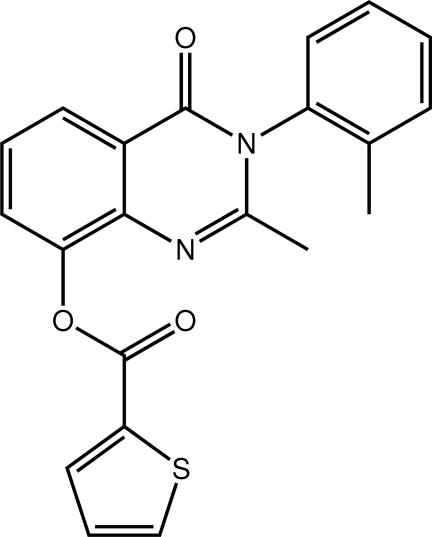


## Experimental

### 

#### Crystal data

C_21_H_16_N_2_O_3_S
*M* *_r_* = 376.42
Monoclinic, 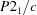

*a* = 5.8031 (1) Å
*b* = 13.4281 (2) Å
*c* = 22.4853 (4) Å
β = 93.115 (2)°
*V* = 1749.57 (5) Å^3^
*Z* = 4
Cu *K*α radiation
μ = 1.86 mm^−1^
*T* = 100 K
0.25 × 0.15 × 0.05 mm

#### Data collection

Agilent SuperNova Dual diffractometer with an Atlas detector
Absorption correction: multi-scan (*CrysAlis PRO*; Agilent, 2011 ▶) *T*_min_ = 0.917, *T*_max_ = 1.000
7119 measured reflections
3582 independent reflections
3119 reflections with *I* > 2σ(*I*)
*R*_int_ = 0.020

#### Refinement

*R*[*F* ^2^ > 2σ(*F* ^2^)] = 0.033
*wR*(*F* ^2^) = 0.091
*S* = 1.03
3582 reflections
260 parameters
34 restraints
H-atom parameters constrained
Δρ_max_ = 0.27 e Å^−3^
Δρ_min_ = −0.27 e Å^−3^

### 

Data collection: *CrysAlis PRO* (Agilent, 2011 ▶); cell refinement: *CrysAlis PRO*; data reduction: *CrysAlis PRO*; program(s) used to solve structure: *SHELXS97* (Sheldrick, 2008 ▶); program(s) used to refine structure: *SHELXL97* (Sheldrick, 2008 ▶); molecular graphics: *ORTEP-3* (Farrugia, 1997 ▶) and *DIAMOND* (Brandenburg, 2006 ▶); software used to prepare material for publication: *publCIF* (Westrip, 2010 ▶).

## Supplementary Material

Crystal structure: contains datablock(s) global, I. DOI: 10.1107/S1600536812006459/gg2075sup1.cif

Structure factors: contains datablock(s) I. DOI: 10.1107/S1600536812006459/gg2075Isup2.hkl

Supplementary material file. DOI: 10.1107/S1600536812006459/gg2075Isup3.cml

Additional supplementary materials:  crystallographic information; 3D view; checkCIF report

## Figures and Tables

**Table 1:** Hydrogen-bond geometry (Å, °)

*D*—H⋯*A*	*D*—H	H⋯*A*	*D*⋯*A*	*D*—H⋯*A*
C9—H9*A*⋯O3^i^	0.95	2.60	3.3472 (16)	136
C13—H13*A*⋯O2^ii^	0.98	2.52	3.4512 (16)	160
C21—H21*C*⋯O2^iii^	0.98	2.53	3.4563 (16)	158

Symmetry codes: (i) 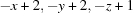; (ii) 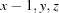; (iii) 
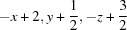
.

**Fig. 1.**
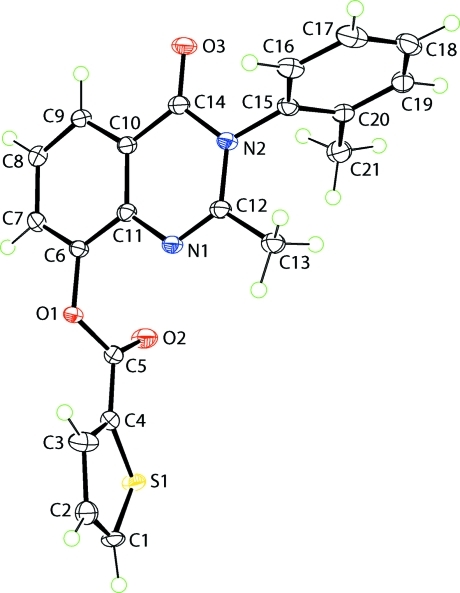
The molecular structure of (I) showing the atom-labelling scheme and displacement ellipsoids at the 50% probability level.

**Fig. 2.**
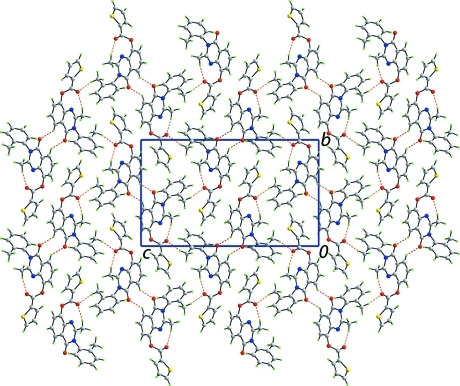
A view in projection down the a axis of the unit-cell contents for (I). The C—H···O interactions are shown as orange dashed lines,

## supplementary crystallographic information

### Comment

Substituted quinazoline-4(3*H*)-ones are known to exhibit biological activity (El-Azab & El-Tahir, 2012; El-Azab *et al.*, 2010; El-Azab *et al.*, 2011; Al-Omary *et al.*, 2010; Al-Obaid *et al.*, 2009; Aziza *et al.*, 1996). In this connection, recently the title compound, (I), a methaqualone analogue, was synthesized and evaluated for its anti-convulsant activity (El-Azab *et al.*, 2010). The crystal structure determination of (I) is reported herein.

In (I), Fig. 1, the 11 atoms comprising the quinazolin-4-one ring are planar [r.m.s. deviation = 0.037 Å]. Both aromatic residues are almost perpendicular to the central plane with the dihedral angles between it and the *N*-bound benzene and thiophenyl rings being 82.22 (5) and 77.05 (13)°, respectively [the dihedral angle = 76.0 (3)° for the minor component of the disordered thiophenyl ring]. There is a twist in the thiophen-2-carboxylate residue as seen in the value of the S1—C4—C5—O2 torsion angle of -18.14 (19)° [161.71 (14)° for the minor component].

In the crystal packing, the molecules are connected into the three-dimensional array by C—H···O interactions involving both carbonyl-O atoms, Fig. 2 and Table 1.

### Experimental

A mixture of 8-hydroxymethaqualone (532 mg, 0.002 *M*) and thiophen-2-carbonyl chloride (307 mg, 0.0021 *M*) in 10 ml pyridine was stirred at room temperature for 9 h. The solvent was removed under reduced pressure, and the residue was triturated with water and filtered. The solid obtained was dried and recrystallized from EtOH. *M*.pt.: 457–459 K. Yield: 94%. ^1^H NMR (500 MHz, CDCl_3_): δ 8.24 (d, 1H, J = 7.5 Hz), 8.10 (d, 1H, J = 3.5 Hz), 7.71 (d, 1H, J = 5.0 Hz), 7.65 (d, 1H, J = 8.0 Hz), 7.50 (t, 1H, J = 8.0 Hz), 7.40–7.36 (m, 3H), 7.22 (t, 1H, J = 8.5 Hz), 7.15 (d, 1H, J = 7.0 Hz), 2.14 (s, 3H), 2.13 (s, 3H) p.p.m.; ^13^C NMR (CDCl_3_): δ 17.4, 24.3, 122.4, 125.1, 126.4, 127.4, 127.7, 127.9, 128.1, 129.6, 131.9, 132.6, 133.7, 135.0, 135.4, 136.8, 141.0, 145.8, 154.9, 160.5, 161.1 p.p.m.; MS (70 eV): m/*z* = 376.

### Refinement

Carbon-bound H-atoms were placed in calculated positions [C—H = 0.95 to 0.98 Å, *U*_iso_(H) = 1.2–1.5*U*_eq_(C)] and were included in the refinement in the riding model approximation. The thiophene ring is disordered over two positions, which are approximately parallel and oppositely orientated. The major component refined to a site occupancy factor = 0.6555 (17). The S–C distances were restrained to 1.71±0.01 Å, the formal C–C single-bond distances were restrained to 1.42±0.01 Å and the formal C–C single bond distances to 1.36±0.01 Å. The isotropic temperature factors of the primed atoms were set to those of the unprimed ones; the anisotropic displacement factors were restrained to be nearly isotropic.

### Crystal data

**Table:**

C_21_H_16_N_2_O_3_S	*F*(000) = 784
*M**_r_* = 376.42	*D*_x_ = 1.429 Mg m^−^^3^
Monoclinic, *P*2_1_/*c*	Cu *K*α radiation, λ = 1.5418 Å
Hall symbol: -P 2ybc	Cell parameters from 3838 reflections
*a* = 5.8031 (1) Å	θ = 3.3–75.8°
*b* = 13.4281 (2) Å	µ = 1.86 mm^−^^1^
*c* = 22.4853 (4) Å	*T* = 100 K
β = 93.115 (2)°	Prism, colourless
*V* = 1749.57 (5) Å^3^	0.25 × 0.15 × 0.05 mm
*Z* = 4	

### Data collection

**Table:**

Agilent SuperNova Dual diffractometer with an Atlas detector	3582 independent reflections
Radiation source: SuperNova (Cu) X-ray Source	3119 reflections with *I* > 2σ(*I*)
Mirror monochromator	*R*_int_ = 0.020
Detector resolution: 10.4041 pixels mm^-1^	θ_max_ = 76.0°, θ_min_ = 3.8°
ω scan	*h* = −7→4
Absorption correction: multi-scan (*CrysAlis PRO*; Agilent, 2011)	*k* = −16→16
*T*_min_ = 0.917, *T*_max_ = 1.000	*l* = −24→28
7119 measured reflections	

### Refinement

**Table:**

Refinement on *F*^2^	Secondary atom site location: difference Fourier map
Least-squares matrix: full	Hydrogen site location: inferred from neighbouring sites
*R*[*F*^2^ > 2σ(*F*^2^)] = 0.033	H-atom parameters constrained
*wR*(*F*^2^) = 0.091	*w* = 1/[σ^2^(*F*_o_^2^) + (0.0489*P*)^2^ + 0.431*P*] where *P* = (*F*_o_^2^ + 2*F*_c_^2^)/3
*S* = 1.03	(Δ/σ)_max_ = 0.001
3582 reflections	Δρ_max_ = 0.27 e Å^−^^3^
260 parameters	Δρ_min_ = −0.27 e Å^−^^3^
34 restraints	Extinction correction: *SHELXL97* (Sheldrick, 2008), Fc^*^=kFc[1+0.001xFc^2^λ^3^/sin(2θ)]^-1/4^
Primary atom site location: structure-invariant direct methods	Extinction coefficient: 0.0022 (2)

### Special details

**Table:**

Geometry. All e.s.d.'s (except the e.s.d. in the dihedral angle between two l.s. planes) are estimated using the full covariance matrix. The cell e.s.d.'s are taken into account individually in the estimation of e.s.d.'s in distances, angles and torsion angles; correlations between e.s.d.'s in cell parameters are only used when they are defined by crystal symmetry. An approximate (isotropic) treatment of cell e.s.d.'s is used for estimating e.s.d.'s involving l.s. planes.
Refinement. Refinement of *F*^2^ against ALL reflections. The weighted *R*-factor *wR* and goodness of fit *S* are based on *F*^2^, conventional *R*-factors *R* are based on *F*, with *F* set to zero for negative *F*^2^. The threshold expression of *F*^2^ > σ(*F*^2^) is used only for calculating *R*-factors(gt) *etc*. and is not relevant to the choice of reflections for refinement. *R*-factors based on *F*^2^ are statistically about twice as large as those based on *F*, and *R*- factors based on ALL data will be even larger.

### Fractional atomic coordinates and isotropic or equivalent isotropic displacement parameters (Å^2^)

**Table:**

	*x*	*y*	*z*	*U*_iso_*/*U*_eq_	Occ. (<1)
S1	0.80579 (11)	0.33540 (5)	0.66390 (3)	0.02055 (19)	0.6555 (17)
S1'	0.4573 (3)	0.40315 (17)	0.57788 (9)	0.021*	0.3445 (17)
C1	0.5762 (7)	0.2591 (3)	0.6445 (2)	0.0198 (7)	0.6555 (17)
H1	0.5558	0.1954	0.6618	0.024*	0.6555 (17)
C1'	0.434 (2)	0.2918 (7)	0.6120 (4)	0.020*	0.3445 (17)
H1'	0.3071	0.2486	0.6037	0.024*	0.3445 (17)
C2	0.4246 (10)	0.2989 (4)	0.60177 (16)	0.0224 (7)	0.6555 (17)
H2	0.2873	0.2676	0.5862	0.027*	0.6555 (17)
C2'	0.6085 (18)	0.2681 (9)	0.6518 (5)	0.022*	0.3445 (17)
H2'	0.6235	0.2076	0.6735	0.027*	0.3445 (17)
C3	0.5057 (6)	0.3945 (3)	0.58431 (19)	0.0279 (9)	0.6555 (17)
H3	0.4297	0.4338	0.5541	0.033*	0.6555 (17)
C3'	0.7640 (14)	0.3487 (6)	0.6557 (3)	0.028*	0.3445 (17)
H3'	0.8951	0.3509	0.6828	0.033*	0.3445 (17)
O1	0.77268 (16)	0.56081 (7)	0.55610 (4)	0.0195 (2)	
O2	0.98652 (18)	0.54819 (7)	0.64289 (4)	0.0266 (2)	
O3	0.71805 (17)	1.00595 (7)	0.57101 (4)	0.0230 (2)	
N1	0.55491 (18)	0.71666 (8)	0.60749 (5)	0.0172 (2)	
N2	0.51713 (18)	0.89074 (8)	0.62219 (5)	0.0171 (2)	
C4	0.7063 (2)	0.42431 (10)	0.61587 (5)	0.0179 (3)	
C5	0.8380 (2)	0.51640 (9)	0.60887 (6)	0.0181 (3)	
C6	0.8539 (2)	0.65744 (9)	0.54682 (6)	0.0177 (3)	
C7	1.0377 (2)	0.67187 (10)	0.51222 (6)	0.0207 (3)	
H7	1.1211	0.6164	0.4984	0.025*	
C8	1.1023 (2)	0.76923 (10)	0.49728 (6)	0.0210 (3)	
H8	1.2292	0.7798	0.4731	0.025*	
C9	0.9813 (2)	0.84904 (10)	0.51782 (6)	0.0194 (3)	
H9A	1.0217	0.9147	0.5067	0.023*	
C10	0.7984 (2)	0.83409 (9)	0.55517 (5)	0.0170 (3)	
C11	0.7318 (2)	0.73712 (10)	0.57045 (5)	0.0165 (3)	
C12	0.4559 (2)	0.79166 (9)	0.63163 (5)	0.0171 (3)	
C13	0.2624 (2)	0.77224 (10)	0.67128 (6)	0.0211 (3)	
H13A	0.2192	0.7018	0.6688	0.032*	
H13B	0.1293	0.8134	0.6586	0.032*	
H13C	0.3119	0.7888	0.7125	0.032*	
C14	0.6798 (2)	0.91853 (10)	0.58113 (6)	0.0177 (3)	
C15	0.4177 (2)	0.96873 (10)	0.65705 (6)	0.0183 (3)	
C16	0.2311 (2)	1.02233 (10)	0.63269 (7)	0.0231 (3)	
H16	0.1759	1.0106	0.5928	0.028*	
C17	0.1259 (3)	1.09295 (11)	0.66693 (8)	0.0287 (3)	
H17	−0.0032	1.1293	0.6507	0.034*	
C18	0.2093 (3)	1.11049 (11)	0.72481 (7)	0.0299 (3)	
H18A	0.1361	1.1584	0.7485	0.036*	
C19	0.3999 (3)	1.05823 (10)	0.74834 (6)	0.0265 (3)	
H19	0.4574	1.0719	0.7879	0.032*	
C20	0.5086 (2)	0.98587 (10)	0.71497 (6)	0.0204 (3)	
C21	0.7130 (2)	0.92851 (11)	0.74099 (6)	0.0256 (3)	
H21A	0.8403	0.9331	0.7142	0.038*	
H21B	0.6699	0.8585	0.7458	0.038*	
H21C	0.7619	0.9566	0.7799	0.038*	

### Atomic displacement parameters (Å^2^)

**Table:**

	*U*^11^	*U*^22^	*U*^33^	*U*^12^	*U*^13^	*U*^23^
S1	0.0238 (4)	0.0159 (3)	0.0216 (3)	−0.0018 (2)	−0.0013 (2)	0.0059 (2)
C1	0.0208 (18)	0.0143 (13)	0.0245 (18)	−0.0067 (11)	0.0046 (12)	0.0032 (12)
C2	0.0250 (11)	0.0231 (13)	0.019 (2)	−0.0022 (10)	−0.0033 (14)	−0.0002 (12)
C3	0.0283 (19)	0.0165 (13)	0.0391 (19)	0.0004 (13)	0.0032 (14)	0.0046 (11)
O1	0.0305 (5)	0.0113 (4)	0.0165 (4)	−0.0025 (4)	0.0001 (4)	0.0004 (3)
O2	0.0319 (5)	0.0215 (5)	0.0255 (5)	−0.0071 (4)	−0.0059 (4)	0.0044 (4)
O3	0.0272 (5)	0.0133 (4)	0.0290 (5)	−0.0008 (4)	0.0054 (4)	0.0034 (4)
N1	0.0209 (5)	0.0134 (5)	0.0172 (5)	−0.0023 (4)	0.0000 (4)	0.0010 (4)
N2	0.0204 (5)	0.0127 (5)	0.0182 (5)	−0.0018 (4)	0.0006 (4)	0.0001 (4)
C4	0.0216 (6)	0.0153 (6)	0.0169 (6)	0.0004 (5)	0.0028 (5)	−0.0005 (5)
C5	0.0222 (6)	0.0144 (6)	0.0179 (6)	0.0019 (5)	0.0030 (5)	0.0000 (5)
C6	0.0255 (6)	0.0124 (6)	0.0150 (6)	−0.0022 (5)	−0.0020 (5)	0.0004 (5)
C7	0.0284 (7)	0.0161 (6)	0.0177 (6)	0.0026 (5)	0.0017 (5)	−0.0021 (5)
C8	0.0256 (6)	0.0212 (7)	0.0163 (6)	−0.0016 (5)	0.0036 (5)	0.0004 (5)
C9	0.0261 (6)	0.0150 (6)	0.0170 (6)	−0.0023 (5)	0.0008 (5)	0.0023 (5)
C10	0.0220 (6)	0.0139 (6)	0.0150 (6)	−0.0016 (5)	−0.0005 (5)	0.0013 (5)
C11	0.0199 (6)	0.0152 (6)	0.0141 (5)	−0.0020 (5)	−0.0019 (5)	0.0013 (5)
C12	0.0201 (6)	0.0138 (6)	0.0169 (6)	−0.0027 (5)	−0.0019 (5)	0.0014 (5)
C13	0.0222 (6)	0.0172 (6)	0.0243 (6)	−0.0028 (5)	0.0035 (5)	0.0006 (5)
C14	0.0196 (6)	0.0151 (6)	0.0183 (6)	−0.0012 (5)	−0.0006 (5)	0.0018 (5)
C15	0.0198 (6)	0.0130 (6)	0.0226 (6)	−0.0026 (5)	0.0049 (5)	−0.0002 (5)
C16	0.0221 (6)	0.0163 (6)	0.0308 (7)	−0.0020 (5)	0.0013 (5)	0.0021 (5)
C17	0.0225 (7)	0.0181 (7)	0.0463 (9)	0.0009 (5)	0.0077 (6)	0.0029 (6)
C18	0.0344 (8)	0.0154 (6)	0.0418 (9)	−0.0030 (6)	0.0196 (7)	−0.0025 (6)
C19	0.0371 (8)	0.0191 (7)	0.0241 (7)	−0.0090 (6)	0.0100 (6)	−0.0023 (5)
C20	0.0250 (6)	0.0154 (6)	0.0212 (6)	−0.0052 (5)	0.0040 (5)	0.0013 (5)
C21	0.0298 (7)	0.0237 (7)	0.0228 (7)	−0.0044 (6)	−0.0031 (5)	0.0010 (6)

### Geometric parameters (Å, º)

**Table:**

S1—C4	1.6904 (14)	C6—C11	1.4036 (18)
S1—C1	1.718 (3)	C7—C8	1.4059 (18)
S1'—C4	1.662 (2)	C7—H7	0.9500
S1'—C1'	1.689 (8)	C8—C9	1.3749 (19)
C1—C2	1.375 (4)	C8—H8	0.9500
C1—H1	0.9500	C9—C10	1.4033 (18)
C1'—C2'	1.351 (8)	C9—H9A	0.9500
C1'—H1'	0.9500	C10—C11	1.4061 (17)
C2—C3	1.430 (5)	C10—C14	1.4641 (18)
C2—H2	0.9500	C12—C13	1.4940 (18)
C2'—C3'	1.408 (13)	C13—H13A	0.9800
C2'—H2'	0.9500	C13—H13B	0.9800
C3—C4	1.389 (4)	C13—H13C	0.9800
C3—H3	0.9500	C15—C16	1.3877 (19)
C3'—C4	1.383 (5)	C15—C20	1.3975 (19)
C3'—H3'	0.9500	C16—C17	1.384 (2)
O1—C5	1.3633 (15)	C16—H16	0.9500
O1—C6	1.4000 (15)	C17—C18	1.384 (2)
O2—C5	1.1995 (16)	C17—H17	0.9500
O3—C14	1.2185 (16)	C18—C19	1.390 (2)
N1—C12	1.2934 (17)	C18—H18A	0.9500
N1—C11	1.3838 (16)	C19—C20	1.3990 (19)
N2—C12	1.3963 (16)	C19—H19	0.9500
N2—C14	1.4061 (17)	C20—C21	1.506 (2)
N2—C15	1.4468 (16)	C21—H21A	0.9800
C4—C5	1.4666 (18)	C21—H21B	0.9800
C6—C7	1.3677 (19)	C21—H21C	0.9800
			
C4—S1—C1	91.48 (16)	C8—C9—C10	120.42 (12)
C4—S1'—C1'	90.3 (4)	C8—C9—H9A	119.8
C2—C1—S1	113.8 (4)	C10—C9—H9A	119.8
C2—C1—H1	123.1	C9—C10—C11	120.37 (12)
S1—C1—H1	123.1	C9—C10—C14	121.00 (11)
C2'—C1'—S1'	115.7 (9)	C11—C10—C14	118.58 (11)
C2'—C1'—H1'	122.1	N1—C11—C6	118.86 (11)
S1'—C1'—H1'	122.1	N1—C11—C10	123.61 (12)
C1—C2—C3	109.4 (4)	C6—C11—C10	117.53 (11)
C1—C2—H2	125.3	N1—C12—N2	123.75 (11)
C3—C2—H2	125.3	N1—C12—C13	118.68 (11)
C1'—C2'—C3'	108.3 (10)	N2—C12—C13	117.56 (11)
C1'—C2'—H2'	125.8	C12—C13—H13A	109.5
C3'—C2'—H2'	125.8	C12—C13—H13B	109.5
C4—C3—C2	113.5 (3)	H13A—C13—H13B	109.5
C4—C3—H3	123.3	C12—C13—H13C	109.5
C2—C3—H3	123.3	H13A—C13—H13C	109.5
C4—C3'—C2'	113.1 (7)	H13B—C13—H13C	109.5
C4—C3'—H3'	123.5	O3—C14—N2	120.93 (12)
C2'—C3'—H3'	123.5	O3—C14—C10	125.26 (12)
C5—O1—C6	117.08 (10)	N2—C14—C10	113.77 (11)
C12—N1—C11	117.32 (11)	C16—C15—C20	121.97 (13)
C12—N2—C14	122.55 (11)	C16—C15—N2	119.17 (12)
C12—N2—C15	119.68 (10)	C20—C15—N2	118.84 (12)
C14—N2—C15	117.73 (10)	C17—C16—C15	119.61 (14)
C3'—C4—C3	106.6 (4)	C17—C16—H16	120.2
C3'—C4—C5	125.4 (4)	C15—C16—H16	120.2
C3—C4—C5	128.00 (19)	C16—C17—C18	119.83 (14)
C3'—C4—S1'	112.4 (4)	C16—C17—H17	120.1
C5—C4—S1'	122.17 (12)	C18—C17—H17	120.1
C3—C4—S1	111.74 (18)	C17—C18—C19	120.16 (14)
C5—C4—S1	120.14 (10)	C17—C18—H18A	119.9
S1'—C4—S1	117.70 (11)	C19—C18—H18A	119.9
O2—C5—O1	123.75 (12)	C18—C19—C20	121.29 (14)
O2—C5—C4	126.32 (12)	C18—C19—H19	119.4
O1—C5—C4	109.90 (11)	C20—C19—H19	119.4
C7—C6—O1	119.68 (12)	C19—C20—C15	117.11 (13)
C7—C6—C11	122.19 (12)	C19—C20—C21	121.05 (13)
O1—C6—C11	117.98 (11)	C15—C20—C21	121.84 (12)
C6—C7—C8	119.61 (12)	C20—C21—H21A	109.5
C6—C7—H7	120.2	C20—C21—H21B	109.5
C8—C7—H7	120.2	H21A—C21—H21B	109.5
C9—C8—C7	119.79 (12)	C20—C21—H21C	109.5
C9—C8—H8	120.1	H21A—C21—H21C	109.5
C7—C8—H8	120.1	H21B—C21—H21C	109.5
			
C4—S1—C1—C2	0.5 (4)	C12—N1—C11—C6	175.58 (11)
C4—S1'—C1'—C2'	0.0 (10)	C12—N1—C11—C10	−3.99 (18)
S1—C1—C2—C3	1.0 (6)	C7—C6—C11—N1	−176.84 (12)
S1'—C1'—C2'—C3'	2.4 (14)	O1—C6—C11—N1	7.56 (17)
C1—C2—C3—C4	−2.6 (6)	C7—C6—C11—C10	2.76 (19)
C1'—C2'—C3'—C4	−4.2 (13)	O1—C6—C11—C10	−172.84 (11)
C2'—C3'—C4—C3	0.2 (9)	C9—C10—C11—N1	179.30 (11)
C2'—C3'—C4—C5	−178.8 (6)	C14—C10—C11—N1	1.97 (18)
C2'—C3'—C4—S1'	4.4 (9)	C9—C10—C11—C6	−0.27 (18)
C2'—C3'—C4—S1	−150 (4)	C14—C10—C11—C6	−177.61 (11)
C2—C3—C4—C3'	−0.2 (6)	C11—N1—C12—N2	0.19 (18)
C2—C3—C4—C5	178.8 (3)	C11—N1—C12—C13	179.29 (11)
C2—C3—C4—S1'	−147 (2)	C14—N2—C12—N1	5.78 (19)
C2—C3—C4—S1	3.0 (4)	C15—N2—C12—N1	−171.94 (12)
C1'—S1'—C4—C3'	−2.5 (6)	C14—N2—C12—C13	−173.32 (11)
C1'—S1'—C4—C3	33 (2)	C15—N2—C12—C13	8.96 (17)
C1'—S1'—C4—C5	−179.4 (4)	C12—N2—C14—O3	175.02 (12)
C1'—S1'—C4—S1	0.4 (4)	C15—N2—C14—O3	−7.22 (18)
C1—S1—C4—C3'	29 (4)	C12—N2—C14—C10	−7.25 (17)
C1—S1—C4—C3	−2.0 (3)	C15—N2—C14—C10	170.51 (11)
C1—S1—C4—C5	−178.2 (2)	C9—C10—C14—O3	3.8 (2)
C1—S1—C4—S1'	2.0 (2)	C11—C10—C14—O3	−178.87 (12)
C6—O1—C5—O2	−12.14 (18)	C9—C10—C14—N2	−173.80 (11)
C6—O1—C5—C4	169.83 (10)	C11—C10—C14—N2	3.51 (16)
C3'—C4—C5—O2	−14.8 (5)	C12—N2—C15—C16	−98.58 (14)
C3—C4—C5—O2	166.4 (3)	C14—N2—C15—C16	83.59 (15)
S1'—C4—C5—O2	161.71 (14)	C12—N2—C15—C20	79.62 (15)
S1—C4—C5—O2	−18.14 (19)	C14—N2—C15—C20	−98.21 (14)
C3'—C4—C5—O1	163.2 (4)	C20—C15—C16—C17	−2.0 (2)
C3—C4—C5—O1	−15.6 (3)	N2—C15—C16—C17	176.13 (12)
S1'—C4—C5—O1	−20.32 (17)	C15—C16—C17—C18	0.8 (2)
S1—C4—C5—O1	159.83 (9)	C16—C17—C18—C19	0.8 (2)
C5—O1—C6—C7	100.68 (14)	C17—C18—C19—C20	−1.3 (2)
C5—O1—C6—C11	−83.61 (14)	C18—C19—C20—C15	0.15 (19)
O1—C6—C7—C8	172.69 (11)	C18—C19—C20—C21	−179.16 (13)
C11—C6—C7—C8	−2.8 (2)	C16—C15—C20—C19	1.50 (19)
C6—C7—C8—C9	0.4 (2)	N2—C15—C20—C19	−176.65 (11)
C7—C8—C9—C10	2.1 (2)	C16—C15—C20—C21	−179.20 (12)
C8—C9—C10—C11	−2.10 (19)	N2—C15—C20—C21	2.65 (19)
C8—C9—C10—C14	175.17 (12)		

### Hydrogen-bond geometry (Å, º)

**Table:**

*D*—H···*A*	*D*—H	H···*A*	*D*···*A*	*D*—H···*A*
C9—H9*A*···O3^i^	0.95	2.60	3.3472 (16)	136
C13—H13*A*···O2^ii^	0.98	2.52	3.4512 (16)	160
C21—H21*C*···O2^iii^	0.98	2.53	3.4563 (16)	158

Symmetry codes: (i) −*x*+2, −*y*+2, −*z*+1; (ii) *x*−1, *y*, *z*; (iii) −*x*+2, *y*+1/2, −*z*+3/2.
